# Chemical, biological and nerve gas attacks: need for education among healthcare personnel and medical students; a Swedish regional cross-sectional web-survey

**DOI:** 10.1186/s12909-024-06488-7

**Published:** 2025-01-05

**Authors:** Albert Gyllencreutz Castellheim, Gustav Persson, Juuso Kuikka, Karol Babinski, Yohan Robinson, Fabian Taube

**Affiliations:** 1 Department of Anesthesiology and Intensive Care Medicine, Institution of Clinical Sciences, Sahlgrenska Academy, University of Gothenburg, Gothenburg, Sweden; 2 Region Västra Götaland, Sahlgrenska University Hospital, Gothenburg, Sweden; 3 Centre for Disaster Medicine, University of Gothenburg, Gothenburg, Sweden; 4 Region Västra Götaland, Norra Älvsborg Hospital, Trollhättan, Sweden; 5 Department of Industrial Engineering and Management, Chalmers University of Technology, Gothenburg, Sweden; 6 Centre for Defense Medicine, Swedish Armed Forces, Gothenburg, Sweden; 7 School of Public Health and Community Medicine, Institute of Medicine, Sahlgrenska Academy, University of Gothenburg, Gothenburg, Sweden; 8 Queen Silvia’s Children Hospital, Behandlingsvägen 7A, Plan 2, 416 50 Gothenburg, Sweden

**Keywords:** Disaster Management, Emergency Preparedness, Professional Education, Undergraduate Medical Education, Biological Warfare, Chemical Warfare

## Abstract

**Background:**

Chemical, biological and nerve gas events have a significant impact on public health, necessitating proper education and training. This study investigated the educational needs as perceived by two groups, frontline healthcare workers and medical students, in relation to chemical, biological, and nerve gas events.

**Methods:**

Three distinct web-based cross-sectional surveys were conducted, one each for chemical, biological, and nerve gas events, with each survey following the same structural format including sections on (a) theoretical knowledge assessment, using multiple-choice questions regarding identification, protection, and treatment, (b) perception of threat, using questions based on a 5-point Likert scale to gauge views on threat/preparedness and (c) perception of existing competency, with questions regarding prior education and the need for additional education and training.

**Results:**

The surveys on chemical, biological, and nerve agents received responses from 44, 36, and 59 participants respectively, comprising both frontline healthcare workers and medical students. The total response rate was approximately 16%. In the theoretical knowledge section of each survey, neither group of participants reached 51% correct answer rate in any of the three surveys. In the section on perception of threat, the percentages of responses in the low half of the Likert Scale were 43.2%, 53.0%, and 96.4% for biological, chemical, and nerve gas surveys, respectively. For the same surveys, 56.8%, 78.7%, and 87.6% of responses were in the middle of the Likert Scale. Regarding perception of competency, 146.2%, 143.1%, and 170.4% of combined responses indicated low existing competency for biological, chemical, and nerve gas surveys, respectively. High and middle ratings for competency were low across all surveys. The perception of need for education were high in the high half and low in the low half of the Likert Scale. The were no statistically significant differences across the sections among the study groups.

**Conclusions:**

The results indicate a widespread tendency to downplay the presence of significant threat and a perception of low existing competency. There is a broad agreement on the need or education and training in managing events of chemical, biological, and nerve gas agents, for frontline healthcare personnel as well as medical students in Sweden.

**Supplementary Information:**

The online version contains supplementary material available at 10.1186/s12909-024-06488-7.

## Background

A Chemical, Biological, Radiological, Nuclear, and Explosive (CBRNE) incident involves potential or actual exposure to chemical (C) [1, 2], biological (B) [3–5], radiological (R) [6], nuclear (N) [7] or Explosive (E) agents on a significant number of people. The term chemical include chemical warfare agents (CW), such as nerve gases and blistering agents (for example mustard gas), as well as agents that may be classed as toxic industrial chemicals (TIC), such as sulfuric acid and hydrogen sulfide. Due to their dual use, some chemicals, such as phosgene and chlorine, may be classed as both TIC and CW.

CBRNE incidents can happen due to natural events, unintentional accidents, or intentional actions, i.e., warfare and terrorist attacks. Illegal CBRNE material production and use persist despite international agreements. Due to potentially severe public health consequences of CBRNE incidents, healthcare personnel need to be prepared to recognize and respond to such incidents promptly and effectively. In the aftermath of a CBRNE incident, hospitals and healthcare facilities may receive large numbers of patients with diverse and complex injuries or exposures. Education and training ensure that first responders [8–11], emergency department physicians and nurses [12, 13], and medical students [14], can provide appropriate care, including protecting themselves and preventing secondary contamination, triage, decontamination, and specialized treatment for such conditions. Furthermore, healthcare personnel should be prepared to implement large-scale medical response in case of mass exposure to CBRN agents.

There are gaps in clinical knowledge as well as in prehospital care [15]. There is also an urgent need to enhance knowledge and awareness among first responders [8]. Different educational modalities have been proposed, e.g., a competency-based blended learning course with enhanced simulation training for frontline healthcare personnel [16], advanced mass-casualty life support (MCLS) course [17, 18], as well as initiatives on education and training programs for nurses and medical students [13, 14]. During the last two decades, various training programs in acute management of CBRNE incidents and mass-casualty have been proposed for civilians and healthcare personnel [10, 12, 19–22].

In the United States, which has been a leader in CBRNE preparedness since the 1950s, there are still notable shortcomings in disaster training courses, despite the existence of a wide array of such programs. These shortcomings include the intended audience, the teaching methods, and the delivery modes, especially with regards to training of healthcare and public health professionals [23]. This highlights the global challenge in developing comprehensive CBRNE education, even in countries with advanced preparedness systems [23]. Since healthcare personnel often serve as coordinators between different response agencies and medical facilities during CBRNE incidents, their ability to communicate effectively and collaborate with other responders can be critical for the outcome of a CBRNE incident. Also, in addition to treating the affected individuals, healthcare personnel do play a vital role in public health management following CBRNE incidents, such as providing public health education to prevent further spread of contamination or disease, but also in mitigating the psychological impact that CBRNE incidents can have on survivors and responders.

However, CBRNE education should extend beyond healthcare personnel to encompass hospital administrators, law enforcement, fire departments, civil defense units, and government agencies. This comprehensive approach is crucial for enhancing public health preparedness [21, 24–27] and the ability to respond effectively to complex and potentially catastrophic CBRNE incidents [16, 28]. The aim is to minimize the impact of such incidents at both the community and national levels.

The aim of the present study was to investigate the perceived need for education among frontline hospital physicians (including both physicians and paramedics) and medical students in handling events of chemical, biological, and nerve gas exposure as acts of war or terror.

## Methods

We utilized a cross-sectional online survey methodology, implementing three distinct structured surveys to address chemical, biological, and nerve gas agents respectively.

### Study setting

At the University of Gothenburg, the research semester is mandatory for all medical students during their second-to-last semester. In the spring of 2023, as their research semester at the University of Gothenburg, three medical students conducted separate research projects focusing on chemical, biological and nerve gas agents, respectively. The study design involved three separate cross-sectional surveys, with each survey assigned to an individual medical student as their research project. These students were responsible for participating in survey design, administering the survey, collecting and analyzing the responses, and writing a comprehensive research report. These individual research projects collectively formed the basis of the current study.

### Ethics approval and consent to participate

According to the Swedish Act on Ethical Review of Research Involving Humans, a completely anonymous survey that does not involve sensitive personal data, physical interventions, or other risks covered by the Act (Sects. 3 and 4), and does not entail a significant intrusion into personal integrity, may be exempt from ethical review as determined by the research principal (Sect. 40 of the Act). Given the voluntary and anonymous nature of our study, the Sahlgrenska Medical Faculty’s Institutional Review Board determined that ethical approval was not necessary under Swedish ethical review laws. The Sahlgrenska Medical Faculty’s Institutional Review Board waived the informed consent. Participants were informed about the study’s purpose, its anonymous and voluntary nature before accessing the survey. Their completion of the survey was considered as implied consent. All methods were performed in accordance with the relevant guidelines and regulations. The online survey was distributed to target groups via course administrators or the respective head of department. To guarantee participant confidentiality, the electronic survey system was configured to be completely anonymous.

### Survey development and administration

To investigate the perceived need for education regarding events with chemical, biological and nerve gas, three surveys were constructed, one for each agent chemical, biological and nerve gas (Supplementary material). All surveys shared identical question sections for maintaining a consistent framework for data collection, including (1) Identification, i.e., correct identification of the agent, (2) Protection, i.e., measures related to self-protection and safeguarding of others, (3) Treatment, i.e., treatment options in case of chemical, biological, or nerve gas attacks, (4) Threat and preparedness, i.e., respondents’ perception of threat and respond preparedness as healthcare personnel, (5) Competency, i.e., respondents’ perception of competency in their own unit and organization, (6) Prior theoretical and practical education, (7) Perception of need for further theoretical and practical education, and (8) Demographics.

The first three sections as well as the demographic section encompassed multiple-choice questions, while the other sections consisted of a 5-point increasing agreement Likert scale ranging from 1 to 5. This rating system allows individuals to express their level of disagreement or agreement with a statement by selecting one of five response options, typically ranging from strongly disagree (score 1) to strongly agree (score 5) and the neutral position placed in the middle of scale (score 3).

The survey development process followed a comprehensive approach, combining methodological rigor with practical insights. Initially, the research team defined clear goals and objectives, ensuring every question served a specific purpose. The process began with a review of relevant literature on survey design, complemented by input from peers and experienced frontline healthcare workers, leveraging their practical insights into CBRNE incidents. The target population was carefully considered, and the language and content were tailored to suit the needs of both healthcare professionals and medical students. This was achieved by incorporating teaching experience in medical education and consulting with senior consultants from the Department of Anesthesiology and Intensive Care at Sahlgrenska University Hospital, who provided specialized expertise. Throughout the development, meticulous attention was paid to the surveys’ length, question order, and visual layout to maximize engagement and data quality. The research team focused on phrasing questions clearly and concisely, breaking down complex concepts when necessary. Facts were incorporated directly into the questions, and a consistent layout was maintained throughout.

The process involved multiple rounds of internal evaluations by the research team to refine question content and structure. This was followed by pilot testing with potential respondents to ensure clarity and relevance of questions. Ethical considerations were also addressed, ensuring respondents understood the survey’s purpose and their right to choose the option “Do not know / Not relevant”. Through this process, the questionnaires were refined to minimize respondent burden and potential biases, aiming to produce high-quality research instruments that effectively assess the perceived educational needs regarding chemical, biological, and nerve gas events among frontline healthcare workers and medical students [29, 30]. The final multiple revised surveys were then administered to the study groups using Mentimeter (https://www.mentimeter.com), which is a digital platform widely adopted within the Swedish academic community for the purpose of conducting web-surveys. The surveys were distributed to the students by course boards, and to healthcare personnel by the heads of hospital departments in March 2023. The respondents were given a two-week window to respond. In certain instances, when feasible, the authors conducted group visits to students and healthcare personnel, encouraging their participation in the web-survey.

### Recruitment

The study was conducted in four major hospitals within the Västra Götaland Region of Sweden, which has a population of about 1.7 million people and holds significant economic and military importance in Western Sweden. All hospitals are in urban areas and were selected due to their prominence in the region and likelihood of being first responders in CBRNE incidents. Sahlgrenska University Hospital, the largest in the study and the only university hospital in the region, offers highly specialized tertiary care for approximately 600,000 people. It is one of the largest hospitals in Northern Europe, with about 17,000 employees and 2,000 beds across three campuses. The other three hospitals, namely Norra Älvsborg County Hospital, Skaraborg Hospital, and Södra Älvsborg Hospital primarily provide secondary care with some specialized services. Each serves a population of approximately 300,000 people. These hospitals were chosen for their capacity to handle major incidents and their diverse staff of healthcare professionals who would be at the forefront of responding to potential CBRNE events. Together, they represent a comprehensive cross-section of the regional healthcare system, encompassing both tertiary and secondary care facilities. The surveys were administered via department heads to emergency, intensive care, and prehospital staff. It warrants emphasis that in the Swedish healthcare system, anesthesiology physicians, with responsibility for anesthesiology departments and intensive care departments, also serve as prehospital personnel alongside paramedics.

The survey on chemical agents was sent to prehospital and certain anesthesia-intensive care chiefs, the survey on biological agents to emergency department chiefs, and the survey on nerve gas agents was sent to those anesthesia-intensive care chiefs who did not receive the survey on C-agents. We distributed the surveys specifically to the departments that would be the first to encounter a particular CBRNE agent. As to students, the three surveys were randomly administered across various semesters during the final two years of medical training at the University of Gothenburg. It was ensured that each cohort was assigned a single, distinct survey.

### Statistics

Descriptive statistics were used for analyzing the first three sections of the surveys as well as the demographics. Mann-Whitney U test was used for comparing study groups regarding the perceived need for education. For sections constructed with multiple-choice questions, the proportion of right versus guess answers was calculated. Post hoc Cronbach’s alpha tests were performed on the sections constructed with 5-LS questions. We also performed a post hoc assessment by evaluating both the uni-dimensionality and the internal consistency, as quantified by Cronbach’s alpha, in the relevant sections. Cronbach’s alpha serves as an index of internal consistency, reflecting the extent to which individual survey items consistently appraise the same latent construct. To authenticate the integrity of Cronbach’s alpha, an estimation of uni-dimensionality is needed. This estimation is scaled from 0 to 1, with a value of 1 signifying complete uni-dimensionality. The absence of uni-dimensionality may suggest a divergence in the construct being measured by the survey items, a factor that must be integrated into the interpretation of Cronbach’s alpha coefficients. In the present study, the threshold for affirming uni-dimensionality was established at 0.5. Furthermore, it is posited that for Cronbach’s alpha must not descend below 0.60.

## Results

A total number of 139 participants (61 females and 78 males) responded to the three surveys. Of these, 36, 44 and 59 participants responded to the survey on biological, chemical, and nerve gas agents respectively. It is estimated that the initial distribution of the surveys encompassed roughly 850 individuals (350 students and 500 combined physicians and paramedics) yielding an overall response rate of approximately 16%. Table 1 illustrates the demography of the participants. As can be seen, it seemed to be a decrease in participation with age. Moreover, there was a noticeable representation of senior consultants in the survey on nerve gas agents (35.6%). A portion of the participants had previous military experience, which was particularly prevalent in the surveys of chemical and nerve gas agents, 31.8% and 22.0% respectively.

**Table 1:** Demography

Variables	Biological agents (*n* = 36)		Chemical agents (*n* = 44)		Nerve gas agents (*n* = 59)	
	Physicians	Students	Physicians + paramedics	Students	Physicians	Students
Male / Female	12 / 6 (37.5 / 18.8)	8/10 (25 / 31.3)	16/2 (36.4 / 4.5)	9/17 (20.5 / 38.6)	25/10 (42.4 / 16.9)	8/16 (13.6 / 27.1)
**Age in years**						
< 25	0 / 0	9 / 28.1	1 / 2.3	18 / 40.9	0 / 0	12 / 20.3
26–35	10 / 31.3	7 / 21.9	2 / 4.5	8 / 18.2	3 / 5.1	10 / 16.9
36–45	3 / 9.4	2 / 6.25	4 / 9.1	0 / 0	9 / 15.3	0 / 0
46–55	3 / 9.4	0 / 0	7 / 15.9	0 / 0	12 / 20.3	2 / 3.4
56 <	2 / 6.25	0 / 0	4 / 9.1	0 / 0	11 / 18.6	0 / 0
**Experience as physician**						
Residents / Foundation trainees	2 / 6.25	-	0 / 0	-	0 / 0	-
Fellows / Specialty trainees	9 / 28.1	−	0 / 0	−	5 / 8.5	−
Attending physicians / Consultants	3 / 9.4	−	1 / 2.3	−	9 / 15.3	−
Senior consultants	4 / 12.5	−	7 / 15.9	−	21 / 35.6	−
**Questionnaire completed by**						
anesthesiology physicians	0 / 0	−	7 / 15.9	−	35 / 59.3	−
emergency medicine physicians	10 / 31.3	−	0 / 0	−	0 / 0	−
Other physicians	8 / 25	-	0 / 0	-	0 / 0	-
Paramedics	0 / 0	-	10 / 22.7	-	0 / 0	-
**Previous military experience**	1 / 3.1	0 / 0	14 / 31.8	1 / 2.3	13 / 22.0	1 / 1.7

Data are presented as count / percent (n / %)

### Evaluation of theoretical knowledge through multiple-choice questions

The section A in Table 2 displays the proportion of right and wrong answers in different sections in the three surveys. The overall wrong answers for biological, chemical, and nerve gas agents were 54.55%, 55.40%, and 75.50% respectively, and overall right answers were 45.45%, 44.60%, and 24.50% respectively. The overall percentage of wrong and right answers for students were 74.40% and 25.60% respectively, while for healthcare personnel it was 69.93% and 30.07% respectively. Physicians performed better than students in all sections in the surveys on biological and nerve gas agents. In the survey on chemical agents, healthcare personnel performed better in the Identification section, while students performed better in Protection and marginally better in Treatment. The section B in Table 2 displays the actual success rates (correct responses) in the theoretical knowledge sections alongside the expected guessed rates if answers were randomly guessed. In the survey on biological agents, the differences between the actual success rates and guessed rates were 0.26, 0.25, and 0.11 for Identification, Protection and Treatment, respectively, meaning that the participants’ actual success rates were higher than the guessed rates across all sections. For the survey on chemical agents, the corresponding values were: 0.25, 0.05, and − 0.09 for Identification, Protection and Treatment, respectively, in which the minus sign indicate a success rate worse than guessing. In the survey of nerve gas agents, the corresponding values were 0.05, 0.01, and − 0.02, respectively, i.e. again with a success rate for Treatment worse than guessing.

**Table 2:** Percentage of correct responses and success rates for multiple-choice questions across biological, chemical, and nerve gas surveys

A: Percentages of wrong and right answers																		
	Biological agents (*n* = 32)						Chemical agents (44 participants)						Nerve gas agents (*n* = 59)					
	Physicians			Students			Healthcare workers			Students			Physicians			Students		
**Multiple Choice sections**	**Wrong**	**Right**		**Wrong**		**Right**	**Wrong**	**Right**		**Wrong**		**Right**	**Wrong**	**Right**		**Wrong**		**Right**
Identification (%)	49.3	50.7		59.8		40.2	60.0	40.0		50.8		49.2	71.4	28.6		79.6		20.4
Protection (self and others) (%)	54.2	45.8		55.4		44.6	70.6	29.4		77.7		22.3	79.4	20.6		85.0		15.0
Treatment (%)	68.2	31.8		78.5		21.5	88.2	11.8		88.0		12.0	72.9	27.1		79.6		20.4
**B: Actual success rates and Guess success rates**																		
	**Biological agents (***n* = **32)**						**Chemical agents (44 participants)**						**Nerve gas agents (***n* = **59)**					
	**Number of questions**		**Actual success rate**		**Guess** **success rate**		**Number of questions**		**Actual success rate**		**Guess** **success rate**		**Number of questions**		**Actual success rate**		**Guess** **success rate**	
Identification	8		0.46		0.20		5		0.45		0.20		9		0.25		0.20	
Protection (self and others)	4		0.45		0.20		5		0.25		0.20		5		0.21		0.20	
Treatment	5		0.31		0.20		2		0.11		0.20		4		0.18		0.20	

Table shows the results of surveys’ knowledge sections measured by multiple-choice sections

### Evaluation of perceptions through likert scale

Below are the results of the perceptions in the three surveys: biological, chemical, and nerve gas agents. The healthcare professionals and students rated the perception questions using 5-point Likert scale. Table 3 shows the percentage of healthcare personnel and students who rated different perception sections across the three surveys. The ratings are in the lower half of the Likert scale (LHLS), which includes Likert scores of 1 and 2, the midpoint of the scale (MLS), which is Likert scores 3 indicating neutrality, and the upper half of the Likert scale (HHLS), which includes Likert scores of 4 and 5. Table 4 shows the combined ratings of healthcare personnel and students in LHLS, MLS, and HHLS across the three surveys for the perception of threat, existing competency, and preparedness.

**Table 3:** Lower half, midpoint, and higher half of Likert scales in Likert scale sections stratified by survey and participant groups

	Biological agents (*n* = 32)				Chemical agents (*n* = 44)				Nerve gas agents (*n* = 59)			
	Physicians		Students		Healthcare workers		Students		Physicians		Students	
**Multiple Choice sections**	**Wrong**	**Right**	**Wrong**	**Right**	**Wrong**	**Right**	**Wrong**	**Right**	**Wrong**	**Right**	**Wrong**	**Right**
Identification (%)	49.3	50.7	59.8	40.2	60.0	40.0	50.8	49.2	71.4	28.6	79.6	20.4
Protection (self and others) (%)	54.2	45.8	55.4	44.6	70.6	29.4	77.7	22.3	79.4	20.6	85.0	15.0
Treatment (%)	68.2	31.8	78.5	21.5	88.2	11.8	88.0	12.0	72.9	27.1	79.6	20.4
**Likert Scale sections**	**LHLS** ^**•**^	**HHLS** ^**••**^	**LHLS**	**HHLS**	**LHLS**	**HHLS**	**LHLS**	**HHLS**	**LHLS**	**HHLS**	**LHLS**	**HHLS**
Perception of threat (%)	11.1	47.2	32.1	53.6	24.2	45.5	28.8	44.1	48.5	15.1	47.9	8.3
Perception preparedness (%)	48.6	22.2	54.6	20.8	70.0	8.0	34.6	30.8	31.2	31.2	20.0	37.1
Perception of existing competency (%)	72.2	8.7	74.0	12.5	64.6	21.5	78.3	10.1	85.4	4.1	85.0	3.3
Prior theoretical and practical education (%)	85.8	1.2	89.7	2.4	75.2	10.7	93.6	4.3	86.1	5.5	94.2	1.5
Perception of need for education (%)	14.2	70.9	19.4	68.4	16.5	62.4	13.3	71.9	25.4	54.4	20.0	63.3
**Likert Scale sections**	**MLS** ^**•••**^		**MLS**		**MLS**		**MLS**		**MLS**		**MLS**	
Perception of threat (%)	41.7		14.3		30.3		42.3		36.4		43.8	
Perception preparedness (%)	29.2		21.4		22.0		24.7		37.6		42.9	
Perception of existing competency (%)	19.0		18.4		13.8		13.5		10.5		11.7	
Prior theoretical and practical education (%)	13.0		7.9		14.1		2.1		8.4		4.2	
Perception of need for education (%)	15.0		12.2		21.2		14.8		20.1		16.7	
^•^ LHLS: Lower half of the Likert scale ; ^**••**^ HHLS: higher half of the Likert scale; ^**•••**^ MLS: Midpoint Likert scale (neutral answers)												

**Table 4:** The combined ratings of LHLS, MLS, and HHLS

Perception of threat	Combined LHLS (%)	Combined HHLS (%)	Combined MLS (%)
Biological agents	43.2	100.8	56.0
Chemical agents	53.0	89.6	78.7
Nerve gas agents	96.4	23.4	87.6
**Perception of preparedness**			
Biological agents	103.2	43.0	37.4
Chemical agents	104.6	38.8	27.3
Nerve gas agents	51.2	68.3	22.2
**Perception of competency**			
Biological agents	146.2	21.2	37.4
Chemical agents	143.1	31.6	27.3
Nerve gas agents	170.4	7.4	22.2

Table 4 shows the combined ratings of healthcare personnel and students in LHLS, MLS, and HHLS across biological, chemical, and nerve gas agents surveys for the perception of threat, existing competency, and preparedness. The maximum possible sum of each row is 200%

### Perception of threat

A higher percentage of both students and healthcare personnel rated the “Perception of threat” in the HHLS, indicating a higher perception of risk. However, in the case of nerve gas agents, the majority of both groups rated in the LHLS (a lower perception of threat). The MLS ratings show that a significant proportion of participants did not strongly lean towards a low or high perception of threat (Table 3).

Table 4 shows, the combined percentages of HHLS, MLS ratings, and LHLS for healthcare personnel and students in biological, chemical, and nerve gas surveys. A maximum possible value in each row of Table 4 is 200%. Please note that combined response percentages can exceed 100% because there are three separate surveys, each potentially containing up to 100% response rate.

### Perception of preparedness

A larger percentage of healthcare personnel rated in LHLS for dealing with chemical agents compared to biological and nerve gas agents. The HHLS ratings (higher perception of preparedness), are relatively low for both groups across all agent types (Tables 3 and 4).

### Perception of competence

In this section, there is an apparent inclination towards the LHLS (perception of low existing competency) in dealing with all three agent types. This is most pronounced for nerve gas agents, where both groups rated LHLS by over 85%. The HHLS and MLS ratings are low across all surveys for both healthcare personnel and students (Tables 3 and 4).

### Prior theoretical and practical education

The vast majority of both students and healthcare personnel express a lack of prior theoretical and practical education in all three surveys, as evidenced by the high LHLS percentages and very low HHLS percentages. The MLS percentages are relatively low, demonstrating that not many are neutral about their level of prior education (Tables 3 and 4).

### Perception of need for education

There are high percentages in the HHLS across all three surveys. The LHLS percentages are relatively low, indicating that few participants feel there is no need for additional education.

The MLS percentages, reflecting a neutral stance, are moderate, suggesting that there remains a segment of respondents who are undecided (Tables 3 and 4).

#### Differences between students and healthcare personnel

Mann-Whitney U test was used for comparisons of conceptions between students and healthcare personnel. There were no significant differences between the two groups in any of the sections (not shown).

#### Uni-dimensionality and Cronbach’s alpha validation

Table 5 presents uni-dimensionality and Cronbach’s alpha validation of the relevant survey sections across the three surveys. The surveys on biological and chemical agents demonstrated suboptimal uni-dimensionality values for perceptions of threat and preparedness, which implies that these surveys do not entirely capture the constructs they intend to measure. The survey on nerve gas agents satisfied the uni-dimensionality requirement and encompassed Cronbach’s alpha values from 0.7 to 0.9, suggesting good to excellent internal consistency and reliability. Most importantly, the section on the perceived need for education, which aligns with the primary objective of the study, displayed consistent internal reliability across all three surveys.

**Table 5:** Uni-dimensionality and Cronbach’s alpha for validation of the three perception sections across the surveys

	Biological agents			Chemical agents			Nerve gas agents		
Sections	n^1^	UD^2^	α^3^	n	UD	α	n	UD	α
Perception of threat / preparedness	6	0.3	0.5	3	0.4	0.4	5	0.5	0.7
Perception of existing competency	7	0.6	0.6	6	0.5	0.6	5	0.8	0.7
Perception of need for education	7	0.6	0.8	6	0.8	0.8	5	1.0	0.9

^1^ n: number of questions; ^2^ UD: Uni-dimensionality; ^3^ α: Cronbach’s α

## Discussion

In the current research, our focus was on biological and chemical agents, including nerve gases. This focus was predicated on the anticipation that acts of war or terrorism utilizing these agents would be more feasible and potentially more likely to be employed by hostile forces, compared to attacks involving nuclear or radioactive materials. This assessment was based on the generally lower technical and resource barriers associated with biological and chemical weapons. Furthermore, the international repercussions and global attention resulting from hostilities involving biological and chemical agents are typically less severe than those associated with nuclear or radioactive incidents. This relative difference in international response could potentially make biological and chemical agents more attractive to hostile actors seeking to avoid maximum global scrutiny or retaliation. Further, we opted to address nerve gases, which are included in chemical agents, as a distinct category to emphasize their potentially catastrophic effects in comparison to other chemical agents, particularly within the context of warfare or terrorist activities. While numerous hazardous chemical agents are utilized across various settings, and some, such as blister agents (including sulfur mustard, nitrogen mustard, and phosgene oxime) and pulmonary agents (like chlorine and phosgene), have been employed in wars and conflicts, their potential for harm is generally more limited compared to nerve gases. To underscore this crucial distinction, we employed two separate surveys for chemical agents: one for chemical agents in general and another specifically for nerve gases. This methodological approach was designed to highlight the varying degrees of threat posed by different chemical agents in conflict scenarios. By segregating nerve gases in our assessment, we aimed to facilitate a more nuanced understanding of the spectrum of chemical threats and their respective implications for preparedness and response strategies.

We found that both students and healthcare personnel possess limited knowledge on identification of chemical, biological and nerve gas agents, as well as protection (self and others), and treatment of the victims. The knowledge on recognizing and safely managing individuals exposed to biological agents was however better than for chemical or nerve gas agents, possibly due to raised awareness due to the COVID-19 pandemic. In the survey on chemical agents, the knowledge level was only marginally above guessing for Identification and Protection, while for Treatment, the actual success rate was below the guessing rate. The survey on nerve gas agents, indicated that participants’ knowledge in Identification and Protection is scarcely better than random chance, and even below the guessing rate for Treatment.

There was an apparent perception of low existing competency (high LHLS) for all three categories of agents. The combination of high LHLS and low HHLS ratings indicate that the entire study group of healthcare personnel and students perceive a significant gap in competency, although the MLS ratings indicate that a considerable number of both students and physicians are neutral in their perception of preparedness. This could possibly reflect ambivalence or uncertainty about their own or other colleagues’ ability (Table 4).

Linked to the perception of low existing competency, a substantial majority of the respondents felt that they lacked sufficient theoretical and practical education, evident by high percentages of low prior education levels (LHLS) and very few reporting high levels of prior education (HHLS). Both study groups reported a need for more education in these areas, with high demand for further training (high HHLS percentages) and with few who believe that additional education is unnecessary (low LHLS percentages). Neutral attitudes (MLS percentages) are less common, indicating that most participants clearly recognize their educational shortcomings and the need for improvement. This is in line with a recently published study on 908 Swedish medical and nursing students’ self-reported knowledge and competence in different disaster medical topics [18]. The paper concluded that the extent of students’ understanding of various disaster medicine aspects, including CBRNE scenarios, correlates with the duration of medical and nursing education received.

There was a pronounced perception towards low threat, especially for nerve agents, although a substantial number of respondents selected the midpoint of the Likert scale (MLS), indicating a neutral position rather than a definitive inclination towards either a low or high perception of threat. The subdued sense of threat might be linked to Sweden’s prolonged peace and neutrality, or to a thorough background in knowledge, education, and training relevant to these areas, as well as self-perceived state of high preparedness and competency. However, in view of Sweden’s current intense discussions on national security, the perception of threat among healthcare workers and students could potentially increase, because of increased political awareness and geopolitical insight. Moreover, a possible increase in perception of threat might not be restricted to merely healthcare personnel and students, but also to the population in general.

The foundation of preparedness and competency for CBRNE incidents in the hospital setting is established in the emergency departments, which should function within a framework of focusing on preparedness, response, decontamination, and personal protective equipment. Preparedness involves organizational, technological, and individual layers, and a systems approach is recommended for managing CBRNE responses [28]. Standardization of this response is vital for improved preparedness [31]. As hospital subsystems, emergency departments, should also develop detailed CBRNE plans. The World Health Organization has provided guidelines since 2011 to assist hospitals in preparing for a range of emergencies and issued further documents on epidemic preparedness and the operation of public health emergency operations centers [32–34]. There are also specific pieces of literature on hospital preparedness for chemical terror attacks [26, 35], biological hazards [25], severe and infrequent threats [24], and emergency preparedness for nerve agents [27].

Preparedness in healthcare is a complex endeavor that integrates multiple key elements, i.e. professional education, hospital preparedness, research, development, continuous training, and global cooperation. Training for healthcare workers should encompass a range of skills, from identifying CBRNE incidents to the effective use of personal protective gear, implementing decontamination processes, and executing medical treatment protocols. Preparedness for emergencies extends beyond the responsibility of emergency departments and hospitals to encompass public health management and civil defense recovery. A 2019 study outlined seven essential components of CBRNE science for successful planning and recovery. These components include basic and clinical sciences, systems modeling and management, strategic planning, managing responses and incidents, fostering recovery and resilience, distilling lessons learned, and promoting ongoing improvement [36].

Medical and tactical team preparedness, with inherent competency, often improves by analyzing real incidents and deriving lessons from past events [18, 37]. A systematic review in 2022 sought to draw lessons from terror incidents in OECD countries since 2001 to bolster such teams’ preparedness. It revealed a pattern of recurring insights throughout the study period, highlighting the importance of not only acquiring new knowledge but also recognizing and applying previously learned lessons to enhance training and readiness initiatives [37].

Our findings underscore the critical necessity for enhancing the knowledge base of healthcare personnel, with a particular emphasis on frontline practitioners. The evident gaps in competence highlight an urgent need for targeted educational interventions. While immediate efforts should focus on current healthcare professionals, a long-term strategy is equally crucial. Integrating comprehensive education on chemical, biological, and nerve gas attacks into medical curricula would cultivate a broad knowledge foundation among future healthcare providers. This approach would gradually elevate the overall competence within the healthcare system. Therefore, we propose a two-pronged approach: immediate training programs for existing healthcare personnel, coupled with curriculum enhancements for medical students. Prioritizing the latter in medical education will yield sustained benefits, ensuring a healthcare workforce well-equipped to address these specialized threats in the future. In 2021, the Swedish National Board of Health and Welfare was tasked by the government to create national education and training plans for disaster medical preparedness and civil defense [38], covering trauma care, management of CBRNE incidents, disaster medicine, and crisis support. In the subsequent year, the Board submitted a comprehensive proposal outlining knowledge and skill objectives for disaster medicine training and exercises for all relevant healthcare personnel categories [39]. The report concludes that the healthcare knowledge and skills outlined in the proposal may be necessary from the very beginning of a healthcare professional’s career and, as such, could be seamlessly integrated into the foundational education provided by Swedish universities and colleges responsible for the basic education of healthcare personnel.

The importance of CBRNE education in regular medical curricula cannot be overstated, particularly in light of recent events that underscore the critical role of healthcare providers in responding to such incidents. Notable examples include the Novichok poisoning in Salisbury, the VX attack in Kuala Lumpur, and alleged chemical weapon use in Ukraine and Syria. These incidents highlight the ongoing relevance of CBRNE preparedness in contemporary healthcare. Moreover, the apparent erosion of norms regarding chemical weapon use and growing concerns about potential attacks in critical geographies such as the Baltics, Scandinavia, and Eastern Europe further emphasize the urgency of this educational need. By addressing these real-world scenarios and geopolitical concerns, we strengthen the argument for enhanced CBRNE training not only in the centers under study but across wider healthcare education systems. This approach would better equip medical professionals to respond effectively to potential CBRNE incidents, thereby improving overall public health security. Currently, Swedish medical students receive a median of 2 h of disaster medicine education, while nursing students receive a median of 4 h of disaster medicine education [18].

The primary strength of this paper lies in its effective illustration of the CBRNEE concept, confirming the reality of CBRNE-related incidents as tangible threats. Another strength lies in utilization of our three distinct surveys, all structured around the same comprehensive question sections, which facilitated a systematic and comparative analysis of responses across different participant groups.

The study’s limitations include the low response rate. The surveys were distributed via email through course boards to students and heads of hospital departments to healthcare personnel. This approach, while efficient, does not allow for precise tracking of how many individuals received, opened, or interacted with the survey link. We estimated the number of individuals who received the emails based on the typical size of student cohorts and the number of healthcare personnel in the targeted departments. However, we cannot accurately determine how many recipients opened the email, clicked on the survey link, or started but did not complete the survey. The response rates we reported (44, 36, and 59 individuals for chemical, biological, and nerve gas surveys respectively, with an overall response rate of about 16%) are based on these estimates of potential recipients. This limitation in determining exact population and sample sizes is common in voluntary, anonymous web-based surveys distributed through institutional channels. While it affects our ability to calculate precise response rates, the data collected still provides valuable insights into the perceived educational needs and competencies regarding chemical, biological, and nerve gas events among our target groups. The low response rate introduces a significant potential for selection bias. It is plausible that respondents were predominantly healthcare personnel and medica students with pre-existing background knowledge in the field of CBNRE. Consequently, the reported levels of competence across various professions may be overestimated in the results, as they likely reflect a subset of participants with higher baseline expertise. This non-response bias could lead to an inflated assessment of overall competence within the target population. The surveys’ results should therefore be interpreted with caution, acknowledging that they may represent the upper bound of competence rather than an accurate cross-sectional view of all potential respondents. Further, the responses we received are sufficiently diverse to represent a range of attitudes and perceptions. However, the low response rate might affect the generalizability of the findings, which mandates follow-up studies or alternative methods to further validate and strengthen the generalizability of our findings. Further, due to time constraints to complete the research before the end of the university semester, the Cronbach’s alpha test for internal consistency was conducted post hoc. Finally, as discussed above, there are some concerns about the precision with which the precision and validity of the current biological and chemical surveys necessitating further refinement.

In conclusion, in the context of CBRNE, this study indicates that the knowledge on chemical, biological and nerve gas agents among frontline healthcare personnel and medical students is limited. A need for increased educational efforts in western Sweden was expressed by the study participants, who also believed that they are less competent in treating victims of incidents with chemical, biological, or nerve gas agents and have an overall low preparedness to respond to these incidents. We conclude that there is an urgent need of cost-effective and time-efficient educational and training programs on chemical, biological, or nerve gas agents for students and frontline healthcare personnel.

## Electronic Supplementary Material

Below is the link to the electronic supplementary material.

Supplementary Material 1

## Data Availability

The datasets used and/or analyzed during the current study are available from the corresponding author on reasonable request.” under declaration section in your main manuscript.

## Declarations

:
