# Supplementary material for: Chemical, biological and nerve gas attacks: need for education among healthcare personnel and medical students; a Swedish regional cross-sectional web-survey
Source: BMC Med Educ. 2025 Jan 5;25:22. doi: 10.1186/s12909-024-06488-7 (PMC11702020; doi:10.1186/s12909-024-06488-7)
Supplement: Supplementary file 1 — Supplementary Material 1 [file 12909_2024_6488_MOESM1_ESM.pdf]

Chemical, Biological and Nerve Gas Attacks: Need for Education among Healthcare Personnel and Medical Students; a Swedish regional cross-sectional web-survey

Albert Gyllencreutz Castellheim, Gustav Persson, Juuso Kuikka, Karol Babinski, Yohan Robinson, Fabian Taube

Chemical agents survey

Chemical weapons: is more education needed?

1 Identification

1. In a shopping center, approximately 50 people have suddenly fallen ill with respiratory distress, somnolence, and nausea. No other symptoms.
What is the most likely cause?
    Chemical terrorism with an unknown blistering chemical weapon
    Chemical attack with blood-chemical weapon
    Chemical attack with lung-chemical weapon
    Chemical attack with radioactive substance
    None of the options
    Do not know

2. 60 people from Haga (in Linnéstaden in Gothenburg) simultaneously seek medical help for respiratory distress, circulatory impact, and high fever. The 10 most seriously ill die within 2 days after admission.
What is the most likely cause of their illness?
    Infection epidemic, unknown agent
    Bioterrorism with microorganism
    Chemical attack with blood-chemical weapon
    Chemical attack with radioactive substance
    None of the options
    Do not know

3. 12 hours ago, several municipal politicians were exposed to a targeted terrorist attack. Now the politicians have raspy breathing and require respiratory support in the ICU.
What is the most likely cause of their illness?
    Infection epidemic, unknown agent
    Chemical attack with blood chemical weapon
    Chemical attack with lung chemical weapon
    Chemical attack with radioactive substance
    None of the options

Do not know

4. Five people in a government building were exposed to a slightly yellowish gas and immediately suffered muscle twitching and vomiting. All of them died within a minute.
What is the most likely cause of the sickness?

Infection epidemic, unknown agent
None of the options
Chemical attack with blood chemical weapon
Chemical attack with lung chemical weapon
Chemical attack with skin chemical weapon
Do not know

5. In a shopping center, a well-known TV journalist had an unknown substance thrown at him. He was immediately affected by respiratory distress and suffered from nosebleeds and facial flushing. All passers-by who tried to help him developed similar symptoms.

What is the most likely cause?
Bioterrorism with microorganism
Chemical attack with lung chemical weapon
Chemical attack with blood chemical weapon
Chemical attack with skin chemical weapon
None of the options
Do not know

2 Protection (self and others)

1. FFP3 is a type of respiratory mask. Against which of the following chemical weapons does FFP3 provide adequate protection?

Mustard gas (blistering chemical weapon)
Cyanogen chloride
Phosgene gas
Sarin
None of the options
Do not know

2. Vinyl gloves are often used in emergency rooms.
Against which of the following chemical weapons do vinyl gloves provide adequate protection?

Mustard gas (blistering chemical weapon)
Cyanogen chloride
Phosgene gas
Sarin
None of the options
Do not know

3. The safety distance from a site of a chemical attack is the distance within which one can stay without protective equipment.
What is the minimum safety distance to a site where chlorine gas has been released in a windless outdoor environment?

at least 10m
at least 100m
at least 500m
at least 1000m
at least 3000m
Do not know

4. A damp cloth pressed over the mouth can be thought to provide some protection in certain situations.
Against which chemical weapon could a damp cloth act as a partial protection so that the exposed person can leave the site without life-threatening exposure?

Cyanogen chloride
Mustard gas
Sarin (nerve gas)
Phosgene
None of the options
Do not know

5. Incident site with a radioactive substance. What protective clothing is required at a minimum to safely conduct a rescue operation?

Respiratory protection FFP3 + plastic aprons + vinyl gloves Respiratory protection
FFP3 + plastic aprons + vinyl gloves + Lead aprons
Fire protective suit
Fire protective suit + Chemical protection suit
None of the options
Do not know

3 Treatment

1. Against which chemical weapon will soap and large amounts of water improve the victim's condition?

Cyanogen chloride
Mustard gas
Sarin (nerve gas)
Phosgene
None of the options
Do not know

2. Chemical attack at Frölunda Torg. Several victims with severe dyspnea and cough after exposure to pulmonary toxic gas (lung chemical weapon).
Which measures will improve the victim's condition?

- Rinse eyes with water, at least 15 minutes
- Remove all clothing
- Intramuscular adrenaline injection (Epipen)
- Intravenous atropine
- None of the options
- Do not know

3. A victim has been exposed to cyanide gas (blood chemical weapon) for 30 minutes. Now the breathing is raspy and it is approximately 15 minutes to the nearest emergency room.
How long a transport can the patient survive?

- 1 minute
- 10 minutes
- 30 minutes
- 1 hour
- 2 hours
- Do not know

4 Perception of threat / preparedness

1. Do you think the risk of chemical attack has increased or decreased over the past 5 years?

- Very likely increased
- Possibly increased
- Neither
- Possibly decreased
- Very likely decreased
- Do not know

2. How likely do you think it is that Västra Götaland could become a target for a chemical attack in the next 5 years?

- Very likely
- Likely
- Neither
- Unlikely
- Very unlikely
- Do not know

3. To what degree do you believe that MSB (Swedish Civil Contingencies Agency) has an overview regarding threat images and the likelihood of chemical attacks?

- To a very high degree
- To a high degree

Partially
To a small degree
To a very small degree
Do not kno

5 Perception of existing competency

1. To what extent do you believe that your classmates/colleagues can identify the initial symptoms that occur upon exposure to a chemical attack?

To a very high degree
To a high degree
Partially
To a small degree
To a very small degree
Do not know

2. To what extent do you believe that your classmates/colleagues have the necessary equipment to be able to treat victims of a chemical attack?

To a very high degree
To a high degree
Partially
To a small degree
To a very small degree
Do not know

3. To what extent do you believe that your classmates/colleagues know how to protect themselves when handling patients who have been exposed to chemical attacks?

To a very high degree
To a high degree
Partially
To a small degree
To a very small degree
Do not know

4. How likely do you think it is that the region's emergency departments have sufficient materials and suitable facilities to handle victims of chemical attacks?

Very likely
Likely
Neither
Unlikely
Very unlikely
Do not know

5. How likely do you believe it is that the Armed Forces can assist in identification and rescue work in a chemical attack?

Very likely
Likely
Neither
Unlikely
Very unlikely
Do not know

6. How likely is it that there are written clinical guidelines (PM) for the management of victims of chemical attacks within VGR (Västra Götalandsregionen)?

Very likely
Likely
Neither
Unlikely
Very unlikely
Do not know

6 Prior theoretical and practical education

1. Swedish Radio broadcasts the program "Beredskap," which is about preparedness for crisis. How often do you listen to the program?

Every week
Every other week
Every month
Less often
Never
Do not know

2. Swedish Radio broadcasts the program "Gräns," which deals with security policy. How often do you listen to the program?

Every week
Every other week
Every month
Less often
Never
Do not know

3. How often do you come into contact with news concerning chemical or biological weapons?

Daily
At least once a week
At least once a month
At least once a year

Less often
Do not know

4. How much theoretical training on chemical attacks have you received during your education and/or service?

A lot
Quite a bit
A little
Very little
None
Do not know

5. How much practical training and clinical simulation on chemical attacks have you received during your education or service?

A lot
Quite a bit
A little
Very little
None
Do not know

6. How much clinical experience do you have with the acute care of victims of chemical attacks (real patients)?

A lot
Quite a bit
A little
Very little
None
Do not know

7. Statement:
I know where to best find updated written information with clinical guidelines/routines to safely treat individuals who have been exposed to a chemical attack.
Do you agree with the statement?

Agree completely
Agree somewhat
Neither disagree nor agree
Somewhat disagree
Disagree completely
Do not know

8. Statement:

I know who to call for safe consultation on protective measures and treatment advice in the event of a chemical attack.
Do you agree with the statement?

Agree completely
Agree somewhat
Neither disagree nor agree
Somewhat disagree
Disagree completely
Do not know

9. Statement:
In a joint effort with the military's medical personnel, rescue work is carried out in the aftermath of a chemical attack. I am certain of who can give me orders.
Do you agree with the statement?

Agree completely
Agree somewhat
Neither disagree nor agree
Somewhat disagree
Disagree completely
Do not know

10. Do you believe that your knowledge and skills regarding chemical attacks are better or worse compared to your colleagues/classmates?

Much better knowledge
Better knowledge
Equally good knowledge
Worse knowledge
Much worse knowledge
Do not know

7 Perception of need for education

1. Statement: Prehospital personnel need more theoretical education on chemical weapons and appropriate actions during chemical attacks.
Do you agree with the statement?

Agree completely
Agree somewhat
Neither disagree nor agree
Somewhat disagree
Disagree completely
Do not know

2. Statement: Prehospital personnel need more practical training on safety routines and proper care of victims of chemical attacks.
Do you agree with the statement?

- Agree completely
- Agree somewhat
- Neither disagree nor agree
- Somewhat disagree
- Disagree completely
- Do not know

3. Statement: Healthcare personnel need interprofessional education with other concerned professional groups (Rescue Services, Police, Armed Forces) on safety routines and proper care of those exposed in major chemical attacks.
Do you agree with the statement?

- Agree completely
- Agree somewhat
- Neither disagree nor agree
- Somewhat disagree
- Disagree completely
- Do not know

4. Statement: The general public needs more education on chemical weapons and correct actions during chemical attacks.
Do you agree with the statement?

- Agree completely
- Agree somewhat
- Neither disagree nor agree
- Somewhat disagree
- Disagree completely
- Do not know

5. Statement: Hospitals in Västra Götaland should prioritize the development of a clear preparedness plan, where the material needs for handling a potential chemical attack are met.
Do you agree with the statement?

- Very high priority
- High priority
- Medium priority
- Low priority
- Very low priority
- Do not know

6. At what point in the career should a theoretical education on the management of chemical attacks be added to your professional category?

Specialist training
Supplementary courses
Late basic education
Early basic education -
Never
Do not know

7. At what point in the career should a practical education with simulation cases involving victims of chemical attacks be added to your professional category?

Specialist training
Supplementary courses
Late basic education
Early basic education
Never
Do not know

Biological agents survey

Bioterrorism and biological warfare: Is more education needed?

1 Identification

1. Which of the following combinations of biological weapons is the most dangerous (Class A)?
    - Creutzfeldt-Jakob and Nipah virus
    - Salmonella and Tularemia
    - Marburg virus and Anthrax
    - Ebola and Ricin
    - Brucellosis and Plague
    - Do not know

2. Which of the following biological weapons can cause serious illness and death?
    - Cryptosporidium
    - Enterotoxin type B
    - C. botulinum toxin
    - V. cholerae
    - C. perfringens
    - Do not know

3. A suitcase containing biological material has been left at the main entrance of Gothenburg Central Station. Several passers-by become acutely ill and need rapid respiratory care. Which biological weapon is most likely?

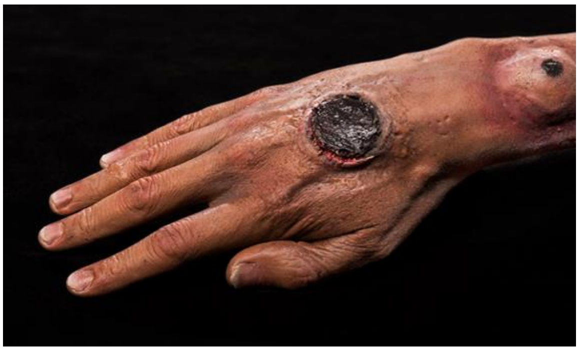

    - Virus
    - Bacteria
    - Bacterial toxins
    - Fungi
    - Amoebas
    - Do not know

4. Given the symptoms of itching vesicles developing into sores without pain, accompanied by fever and cough, and the suspicion of bioterrorism, the most likely biological weapon would be:
Variola major (the virus that causes smallpox)

- Brucella
- Ricin toxin
- Nephropathia epidemica
- Bacillus anthracis (the bacterium that causes anthrax)
- Do not know

5. Eight protesters have developed symptoms including high fever, headache, and general muscle pain over three days. Many have dark red vomiting, hoarseness, and are spitting blood. See picture for skin presentation.

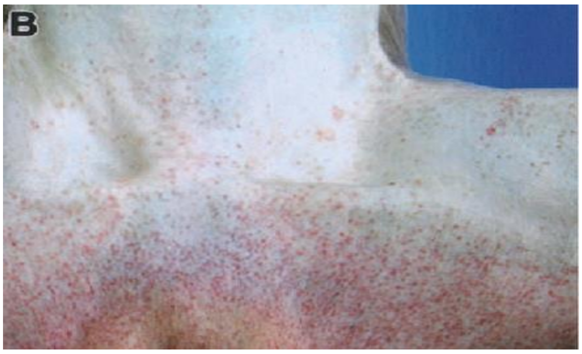

Which biological weapon is most likely?

- Glanders
- Ebola virus
- EHEC
- Shigella
- West Nile fever (West Nile Virus)
- Do not know

6. The image shows a 5-year-old boy with muscle pain and a skin rash. Seven preschool mates have developed the same symptoms. According to the father, it started after the son opened a threatening letter addressed to the father who works in civil defense.

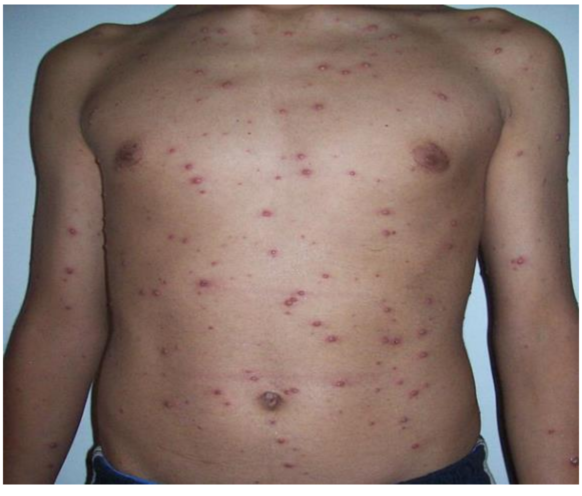

Which biological agent do you think the child has been exposed to?"

Bacillus anthracis
Varicella-Zoster virus
Lassa virus
Yersinia pestis
Variola major
Do not know

7. A dozen journalists in a newspaper editorial office fall ill simultaneously with facial paralysis, shortness of breath, and varying degrees of paralysis. Five die. No findings on blood tests, CT brain, and cerebrospinal fluid.

Which biological weapon is most likely?

Nipah virus
Poliovirus
Japanese encephalitis
C. botulinum toxin
C. perfringens toxin
Do not know

8. Five customs officers seek medical attention on the same day with high fever, headache, and nausea. See picture for the wounds. History of insect bites in all after a mission where they inspected a suspicious container in the port.

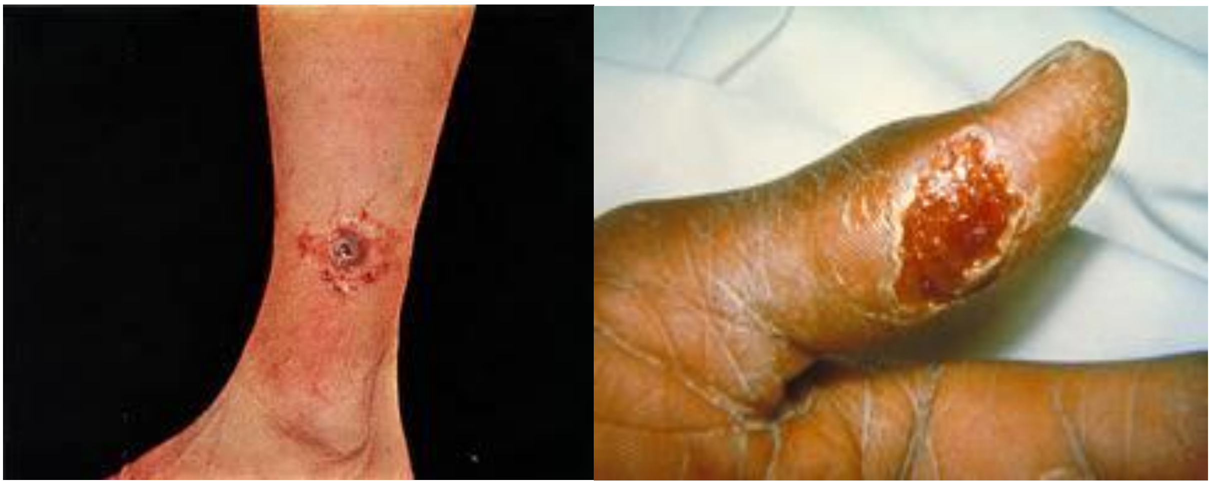

Which biological weapon is most likely?

Borrelia burgdorferi
Francisella tularensis
Staphylococcus aureus
Yersinia pestis
Diphtheria
Do not know

2 Protection (self and others)

1. An FFP3 mask (tight respiratory protection), face shield, protective apron, and gloves, as well as post-exposure prophylaxis with antibiotics, are needed when taking care of victims to certain biological weapons.
Which of the following biological weapons might require these aids?

- Tularemia
- Brucellosis
- Anthrax
- C. perfringens
- Salmonella
- Do not know

2. Plague caused by Yersinia pestis is classified as a biological weapon.
What is the most common route of transmission for plague?

- Urine/feces
- Blood
- Droplet infection (cough)
- Bites from infected fleas
- Wound secretion
- No good answer to the question

3. In a hospital, several cases of viral hemorrhagic fever have been diagnosed with laboratory tests.
Which of the following infection control options is best?

- No action as the disease is not transmissible between humans
- Shared room on the ward with special hygiene routines
- Private room on the ward
- Isolation ward
- High-level isolation ward
- Do not know

4. The effectiveness of a protective mask against airborne transmission can be affected by facial hair.
How does stubble, compared to a clean-shaven face, affect the mask's protection factor?

- Just as good
- 10% worse
- 25% worse
- 50% worse
- 90% worse
- Do not know

3 Treatment

1. Plague caused by Yersinia pestis is classified as a biological weapon.
What is the most important treatment for plague?

- Antivirals
- Antibiotics
- Symptomatic treatment with IV fluids and oxygen
- Y. pestis toxin antibodies
- Antifungals
- Do not know

2. Ricin is a toxin that occurs naturally in a special kind of beans and is classified as a bioweapon.
What is the most important treatment for Ricin toxin exposure?

- Antibodies against ricin toxin
- IV cortisone
- Flush the toxin from exposed body parts
- Post-exposure prophylaxis with antibiotic tablet
- Dialysis
- Do not know

3. Viral hemorrhagic fever is a serious disease classified as a bioweapon.
Which of the following statements is most accurate for viral hemorrhagic fever?
A. Ribavirin is an effective treatment and reduces mortality.
B. There is an effective vaccine that has stopped outbreaks in endemic areas.
C. Prophylactic antibiotic treatment for gastrointestinal perforation is important in suspected cases.
D. Human antibodies are widely used, are readily available, and reduce mortality.
E. There is only symptomatic treatment.

- A
- B
- C
- D
- E
- Do not know

4. Botulism is a serious illness caused by a toxin that attacks the nervous system and causes muscle paralysis, respiratory distress, and possibly death. C. botulinum toxin is classified as a bioweapon.
What is the most important treatment for botulism?

- Cholinesterase inhibitors
- Antibodies against botulinum toxin
- Atropine
- Antibiotics

Plasmapheresis
Do not know

5. Tularemia, also known as rabbit fever, is a vector-borne zoonosis caused by the bacterium F. tularensis and can be transmitted between animals and humans. Tularemia is classified as a bioweapon.
What is the most important treatment for tularemia?
Penicillin V
Ciprofloxacin
Antibodies against F. tularensis toxin
Paracetamol
Plasmapheresis
Do not know

4 Perception of threat / preparedness

1. Do you think the risk of bioterrorism attacks has increased or decreased over the past 5 years in Sweden?
Increased significantly
Increased
Neither nor
Decreased
Decreased significantly
Do not know

2. How likely do you believe it is that the Västra Götaland Region (VGR) will be subjected to a bioterrorism attack within the next 5 years?
Very likely
Likely
Neither nor
Unlikely
Very unlikely
Do not know

3. How good do you think the general public's knowledge is about potential bioterrorism attacks?
Very good knowledge
Good knowledge
Moderate knowledge
Poor knowledge
Very poor knowledge
Do not know

4. How likely do you believe it is that the Swedish Armed Forces have a comprehensive overview of threats from biological warfare?

Very likely
Likely
Neither nor
Unlikely
Very unlikely
Do not know

5. How likely do you believe it is that the Swedish Security Service (SÄPO) has a comprehensive overview of threats from bioterrorism?

Very likely
Likely
Neither nor
Unlikely
Very unlikely
Do not know

6. How likely do you believe it is that the Swedish Civil Contingencies Agency (MSB) has a preparedness plan aimed at the public?

Very likely
Likely
Neither nor
Unlikely
Very unlikely
Do not know

5 Perception of existing competency

1. Do you believe that your colleagues/classmates can identify victims of a bioterrorism attack (for example, Lassa fever and smallpox)?

To a very high degree
To a high degree
Neither nor
To a low degree
Not at all
Do not know

2. Do you believe that your colleagues/classmates can treat victims of a bioterrorism attack (for example, botulism or anthrax)?

To a very high degree
To a high degree

Neither nor
To a low degree
Not at all
Do not know

3. Do you believe that your colleagues/classmates can protect themselves and others when handling victims of a bioterrorism attack (for example, botulism or anthrax)?

To a very high degree
To a high degree
Neither nor
To a low degree
Not at all
Do not know

4. To what extent do you believe that there is already staff with good knowledge and readily available clinical routines at the region's emergency departments for handling bioterrorism attacks?

To a very high degree
To a high degree
Neither nor
To a low degree
Not at all
Do not know

5. To what extent do you believe that the region's emergency departments have enough materials and suitable facilities to handle medium-sized bioterrorism attacks (such as rickettsia or Marburg virus)?

To a very high degree
To a high degree
Neither nor
To a low degree
Not at all
Do not know

6. How likely do you believe it is that the laboratory at your hospital has sufficient competence to identify the most common bioweapons?

Very likely
Likely
Neither nor
Unlikely
Very unlikely
Do not know

7. What proportion of victims do you believe the region's combined intensive care capacity is capable of handling after a bioterrorism attack on a major water reservoir in Gothenburg?

None
Less than half
Half
More than half
All
Do not know

6 Prior theoretical and practical education

1. How closely do you follow the Swedish Radio program "Beredskap" that deals with security policy?

Every week
Every other week
Every month
Less often
Never
Do not know

2. How closely do you follow the Sveriges Radio program "Gräns" which is about security policy?

Every week
Every other week
Every month
Less often
Never
Do not know

3. Estimate how often you come into contact with news concerning chemical or biological weapons/terrorist attacks.

Daily
At least once a week
At least once a month
At least once a year
Less often
Do not know

4. How much theoretical education on biological attacks have you officially received during your medical training and/or service?

A lot
Quite a bit
A little
Very little

None
Do not know

5. How much practical training and clinical simulation of biological attacks have you received during your medical education and/or service?
A lot
Quite a bit
A little
Very little
None
Do not know

6. How much clinical experience do you have with the acute care of victims of biological attacks (real patients)?
A lot
Quite a bit
A little
Very little
None
Do not know

7. Statement: I know who to contact for secure consultation regarding protective measures and treatment advice in the event of a biological attack.
Do you agree with the statement?
Strongly agree
Partially agree
Neither disagree nor agree
Partially disagree
Strongly disagree
Do not know

8. Statement: I know where to best find updated written information with clinical guidelines/procedures for safely treating individuals exposed to biological attacks.
Do you agree with the statement?
Strongly agree
Partially agree
Neither disagree nor agree
Partially disagree
Strongly disagree
Do not know

9. Do you believe that your knowledge and skills regarding biological attacks are better or worse compared to your colleagues/classmates?

Much better knowledge
Better knowledge
Equally good knowledge
Worse knowledge
Much worse knowledge
Do not know

7 Perception of need for education

1. Statement: Doctors need more theoretical education about bioweapons and appropriate measures in the event of a biological attack.
Do you agree with the statement?

Strongly agree
Partially agree
Neither disagree nor agree
Partially disagree
Strongly disagree
Do not know

2. Statement: Doctors need more practical training on security procedures and proper care of biological attack victims.
Do you agree with the statement?

Strongly agree
Partially agree
Neither disagree nor agree
Partially disagree
Strongly disagree
Do not know

3. Statement: Healthcare personnel need interprofessional education with other concerned professional groups (Fire Department, Police, Armed Forces) on security procedures and proper care of those exposed in major biological attacks.
Do you agree with the statement?

Strongly agree
Partially agree
Neither disagree nor agree
Partially disagree
Strongly disagree
Do not know

4. Statement: The general public needs more education about bioweapons and correct actions to take in the event of a biological attack.
To what extent do you agree with the statement?

Strongly agree
Partially agree
Neither disagree nor agree
Partially disagree
Strongly disagree
Do not know

5. Statement: Hospitals in Västra Götaland should prioritize the creation of a clear contingency plan that addresses the material needs for handling a potential biological attack.
To what degree do you agree with the statement?

Very high priority
High priority
Medium priority
Low priority
Very low priority
Do not know

6. At what point in a doctor's professional career should a theoretical education about bioterrorism be placed? Before or after internship/specialist training?

Never
Undergraduate education
During internship
During specialist training
For specialists
Do not know

7. When in a doctor's professional career should practical training about bioterrorism be placed? Before or after internship/specialist training?

Never
Undergraduate education
During internship
During specialist training
For specialists
Do not know

Nerve gas agents survey

Nerve agents: Do we need more education?

1 Identification

1. Which option best describes the characteristic features of a pure nerve agent?
Sharply gasoline-scented, invisible vapors

Odorless, white vapors
Smell of rotten eggs, invisible vapors
Odorless, invisible vapors
Sharply gasoline-scented, white vapors
Do not know

2. What is correct regarding nerve agents and the enzyme cholinesterase (acetylcholinesterase?
Inhibits cholinesterase permanently

Inhibits cholinesterase partially
Synergistic action that increases enzyme activity
Cholinesterase metabolizes nerve agents into toxic metabolites
Nerve agents are not associated with cholinesterase
Do not know

3. During a terrorist attack, a container with sarin gas is thrown through the window into a newspaper editorial office; five journalists are in the office. The container explodes and the entire room is filled with aerosol and nerve gas vapor. The journalists manage to get out after 5 seconds of heavy exposure.
What is the expected survival time for the journalists without medical treatment?

Less than 5 minutes
Between 5 to 20 minutes
Between 20 to 60 minutes
Between 1 to 3 hours
More than 3 hours
Do not know

4. A masked perpetrator smashes two glass bottles close to each other at a public place. Shortly thereafter, the surrounding civilians are affected by the released nerve agent.
Which nerve agent is it most likely?

Tabun
VX

Soman
Sarin
BZ
Do not know

5. 5 employees at an emergency department have been exposed to sarin gas vapors during an attack on a hospital in the Västra Götaland region.
Which symptom usually appears first with this type of nerve gas exposure?

Miosis
Nausea
Mydriasis
Muscle fasciculations
Itching
Do not know

6. During an ongoing rescue operation after a nerve gas attack, one of your colleagues accidentally comes into contact with a contaminated surface (contaminated by nerve gas.
Which symptom usually appears first with skin exposure to nerve gas?

Miosis
Nausea
Mydriasis
Muscle fasciculations
Itching
Do not know

7. What effect does nerve gas exposure have on the airways?

Increased serous secretion, dilation
Increased mucous secretion, constriction
Decreased secretion, dilation
Increased mucous secretion, dilation
Decreased secretion, constriction
Do not know

8. All of the following options, with one exception, are symptoms caused by the effect of nerve agents on the central nervous system.
Which option is the exception?

Facial paralysis
Epileptic seizures
Confusion

Apnea (reduced respiratory drive)
Psychomotor agitation
Do not know

9. In a terrorist attack at Landvetter airport, approximately 200 travelers have been exposed to an unidentified nerve agent, many with only mild symptoms.
Is there any clinical tool to objectively assess the severity of nerve gas exposure?

Nerve gas concentration in exhaled air
Specific blood tests
Calculate ASAT/ALAT ratio
Computer tomography (brain); high intracranial pressure
Only clinical symptoms
Do not know

2 Protection (self and others)

1. Which action should be performed first in the decontamination (bodily cleansing) of those exposed to nerve gas?

Rinse eyes with water for at least 15 minutes
Remove all clothing
The exposed should wash their hands if they have touched contaminated surfaces
The exposed are to wait outdoors for 30 minutes, the nerve agent is volatile and creates toxic vapor
None of the above
Do not know

2. During a lecture on abortion at Sahlgrenska, a hundred people (lecturers and listeners) are exposed to a nerve gas attack. 2 minutes later, several of the affected arrive at the emergency room.
In close contact with the exposed, what is the minimum level of personal protective equipment that should be worn by the medical staff?

No protective equipment is needed
Latex gloves, apron, and tight respirator (FFP3)
Double latex gloves, long-sleeved plastic coat with hood, and full-face gas mask
Full-body plastic suit with boots, double latex gloves, and full-face gas mask
None of the above options provide sufficient protection
Do not know

3. Sarin gas has been released in a square during a demonstration.
Which of the following pieces of advice is INAPPROPRIATE to send out to the nearby public?

Lock yourself and your family in a windowless room
Turn off fans, ventilation, and air heating systems
Seal all door and window cracks with plastic and paper
Do not drink tap water
None of the above is inappropriate
Do not know

4. Sarin gas has been released in a square during a demonstration. The rescue work on the site is completed.
How long should one expect the weather-exposed environment to be contaminated and dangerous to be in without protective equipment?

Less than 1 day
1 to 3 days
3 days to 1 week
1 to 3 weeks
Longer than 3 weeks
Do not know

5. The nerve agent VX has been dispersed in a shopping center. The rescue work on site is completed.
How long is the environment contaminated and dangerous to be in without protective equipment?

Less than 1 day
1 to 3 days
3 days to 1 week
1 to 3 weeks
Longer than 3 weeks
Do not know

3 Treatment

1. A person exposed to vapors of the nerve agent sarin develops eye symptoms, increased saliva production, and a slight pressure sensation in the chest after five minutes. This is classified as mild exposure.
Which of the following is the best initial medication treatment?

Atropine 2 mg intravenously
EpiPen. Adrenaline 0.5 mg intramuscularly
Active expectancy

Diazepam 10 mg orally
Pralidoxime 100 mg intravenously
Do not know

2. A severe symptom picture after exposure to nerve gas includes blurred vision, diarrhea, vomiting, loss of bladder control, muscle twitches, and dyspnea.
Which of the following is the best combination of medications to administer for an untreated severe symptom picture?

Atropine 6 mg + Adrenaline 1 mg
Pralidoxime 100 mg + Adrenaline 1 mg
Atropine 6 mg + Diazepam 10 mg
Adrenaline 1 mg + Diazepam 10 mg
Pralidoxime 100 mg + Atropine 2 mg
Do not know

3. During a rescue operation after a nerve gas attack, your colleague accidentally makes skin contact with a surface contaminated by VX. You immediately initiate medication treatment for mild exposure. Four minutes later, your colleague's saliva production increases.
Is this reason enough to increase the dosage of the medication?

No. The condition is still classified as mild exposure
No. Your colleague will survive, conserve resources
No. Increased dosage is given only in case of dyspnea or apnea
Yes. The degree of secretion guides treatment
Yes. Increased dosage should always be given upon symptom progression after initial treatment
Do not know

4. A politician who has been exposed to the nerve agent tabun is being cared for in the ICU. Correct medication treatment and respiratory care were initiated 2 hours ago. Good prospects for the patient's survival.
How long will the patient likely require respirator-assisted breathing?

Less than 6 hours
6 to 24 hours
1 to 3 days
3 to 7 days
Longer than 7 days
Do not know

4 Perception of threat / preparedness

1. How great do you assess the risk to be that a nerve gas attack/terrorist act would occur in Västra Götaland within the next 20 years?

Very likely
Likely
Neither nor
Unlikely
Very unlikely
Do not know

2. Do you assess that the risk of a nerve gas attack/terrorist act in Västra Götaland has increased or decreased over the past 20 years?

The risk has increased significantly
The risk has increased
Neither increased nor decreased
The risk has decreased
The risk has decreased significantly
Do not know

3. MSB is the abbreviation for the Swedish Civil Contingencies Agency.
To what extent do you believe that the MSB has an accurate picture of a possible nerve gas attack by a foreign power or a terrorist act?

To a very high degree
To a high degree
Partially
To a small degree
To a very small degree
Do not know

4. SÄPO is the abbreviation for the Swedish Security Service.
To what extent do you believe that SÄPO has an accurate picture of a possible terrorist act with nerve gas?

To a very high degree
To a high degree
Partially
To a small degree
To a very small degree
Do not know

5. MUST is the abbreviation for the Swedish Military Intelligence and Security Service.
To what extent do you believe that MUST has an accurate picture of a possible nerve gas attack from a foreign power?

To a very high degree
To a high degree
Partially
To a small degree
To a very small degree
Do not know

5 Perception of existing competency

1. To what extent do you believe your classmates/colleagues can identify nerve agents and the initial symptoms that occur upon exposure?

To a very high degree
To a high degree
Partially
To a small degree
To a very small degree
Do not know

2. To what extent do you believe your classmates/colleagues have the knowledge, experience, and equipment to treat victims after a nerve gas attack?

To a very high degree
To a high degree
Partially
To a small degree
To a very small degree
Do not know

3. To what extent do you believe your classmates/colleagues have the knowledge, experience, and equipment to protect themselves and others from exposure when handling patients who have been subjected to nerve gas?

To a very high degree
To a high degree
Partially
To a small degree
To a very small degree
Do not know

4. To what extent do you believe that the region's emergency departments have sufficient materials and suitable facilities to handle victims in the event of a nerve gas attack?

To a very high degree
To a high degree
Partially
To a small degree
To a very small degree
Do not know

5. In the event of a terrorist attack at Gothenburg Central Station, the majority of those affected survive the transport to the hospital.
What proportion of those severely affected do you think the region's combined intensive care capacity is capable of caring for and treating?

All
Most
About half
Less than half
None
Do not know

6 Prior theoretical and practical education

1. How closely do you follow the Swedish Radio program "Beredskap" that deals with security policy?

Every week
Every other week
Every month
Less often
Never
Do not know

2. How closely do you follow the Swedish Radio program "Gräns" that deals with security policy?

Every week
Every other week
Every month
Less often
Never
Do not know

3. Estimate how often do you come into contact with news concerning chemical or biological weapons?

Daily
At least once a week
At least once a month
At least once a year
Less often
Do not know

4. How much theoretical education about nerve gas attacks have you received during your medical training and/or service?

A lot
Quite a bit
A little
Very little
None
Do not know

5. How much practical training and clinical simulation of nerve gas attacks have you received during your medical training and/or service?

A lot
Quite a bit
A little
Very little
None
Do not know

6. How much clinical experience do you have with the acute management of victims of nerve gas attacks (real patients)?

A lot
Quite a bit
A little
Very little
None
Do not know

7. Statement: I know whom to contact for safe consultation regarding protective measures and treatment advice during nerve gas attacks.
Do you agree with the statement?

Agree completely
Agree somewhat
Neither disagree nor agree
Somewhat disagree
Disagree completely
Do not know

9. Statement: I know where to find the most up-to-date written information with clinical guidelines/routines for safely treating individuals exposed to nerve gas attacks.
Do you agree with the statement?

Agree completely
Agree somewhat
Neither disagree nor agree
Somewhat disagree
Disagree completely
Do not know

7 Perception of need for education

1. Statement: Physicians need more theoretical education on nerve agents and appropriate actions in the event of nerve gas attacks.
Do you agree with the statement?

Agree completely
Agree somewhat
Neither disagree nor agree
Somewhat disagree
Disagree completely
Do not know

2. Statement: Physicians need more practical training on safety procedures and correct management of victims of nerve attacks.
Do you agree with the statement?

Agree completely
Agree somewhat
Neither disagree nor agree
Somewhat disagree
Disagree completely
Do not know

3. Statement: Healthcare personnel need interdisciplinary training with other concerned professional groups (the Fire Service, Police, Armed Forces) on safety procedures and correct management of those exposed during major nerve gas attacks.
Do you agree with the statement?

Agree completely
Agree somewhat
Neither disagree nor agree
Somewhat disagree
Disagree completely
Do not know

4. Statement: The general public needs more education about nerve agents and correct actions in the event of a nerve gas attack.
Do you agree with the statement?

Agree completely
Agree somewhat
Neither disagree nor agree
Somewhat disagree
Disagree completely
Do not know

5. Statement: There is a need for more theoretical education among the general public about nerve agents and correct actions in the event of a nerve gas attack.
Do you agree with the statement?

Agree completely
Agree somewhat
Neither disagree nor agree
Somewhat disagree
Disagree completely
Do not know

6. Statement: Hospitals in Västra Götaland should prioritize the design of a clear contingency plan, where the material needs for handling a potential (bio/chem/nerve attack) are met.
Do you agree with the statement?

Agree completely
Agree somewhat
Neither disagree nor agree
Somewhat disagree
Disagree completely

Do not know

7. At which stage in the professional career should theoretical education about nerve agents be placed in the medical profession?

Training for specialists
Residency training (ST)
Internship/Basic training (AT/BT)
Clinical semesters of medical program
Preclinical semesters of medical program
As an elective course in the new medical program
Do not know

8. At which stage in the career should practical training with simulation cases of nerve gas victims be placed in the medical profession?

Training for specialists
Residency training (ST)
Internship/Basic training (AT/BT)
Clinical semesters of medical program
Preclinical semesters of medical program
As an elective course in the new medical program
Do not know

Demographics (for all three surverys)

Gender
Male / Female / Prefer not to answer

Age
25 or younger / 26-35 / 36-45 / 46-55 / 56 or older / Prefer not to answer

What is your workplace?
SU (Sahlgrenska, Mölndal, Östra) / SÄS / NÄL / SkaS / NU / Medical student at GU / Prefer not to answer

Which professional group do you belong to?
Resident doctor / Specialist in anesthesia / Specialist in emergency medicine / Senior consultant in anesthesia / Senior consultant in emergency medicine / Medical student at GU / Prefer not to answer

Previous military training?

No / The H48 course for healthcare personnel / Basic training (Conscription) / Basic training + additional education in military medicine / Officer training / Prefer not to answer
